# The versatile role of YidC in membrane protein biosynthesis and quality control

**DOI:** 10.1016/j.bbamcr.2025.119956

**Published:** 2025-04-10

**Authors:** Mehmet Caliseki, Christiane Schaffitzel, Burak Veli Kabasakal

**Affiliations:** a Turkish Accelerator and Radiation Laboratory, Ankara 06830, Türkiye; b Department of Molecular Biology, Genetics and Bioengineering, Graduate School of Engineering and Natural Sciences, Sabanci University, Istanbul 34420, Türkiye; c School of Biochemistry, University of Bristol, 1 Tankard’s Close, Bristol BS8 1TD, UK

**Keywords:** YidC, Membrane protein biosynthesis, Membrane protein quality control, Membrane protein insertion, Insertase, Chaperone

## Abstract

Membrane proteins are essential for bacterial survival, facilitating vital processes such as energy production, nutrient transport, and cell wall synthesis. YidC is a key player in membrane protein biogenesis, acting as both an insertase and a chaperone to ensure proper protein folding and integration into the lipid bilayer. Its conserved structure and adaptability enable it to mediate co-translational and post-translational protein insertion into the membrane through both Sec-dependent and Sec-independent pathways. In addition to facilitating protein insertion, YidC collaborates with FtsH in protein quality control, preventing the accumulation of misfolded proteins that could impair cellular function. This important relationship between YidC and FtsH is poorly understood, and there is a need for further investigation into their collaboration. Understanding how YidC and FtsH coordinate their roles could provide valuable insights into the links between bacterial membrane protein biogenesis and quality control pathways. Moreover, given its central functions, YidC represents a potential target for antimicrobial development. Small molecules disrupting its function in protein folding and insertion, hold promise. However, achieving bacterial specificity without impacting eukaryotic homologs remains a challenge. Here, we review our current understanding of YidC’s structure, molecular function in membrane protein biogenesis and quality control, known interactions and its therapeutic potential.

## 1 Introduction

Membrane proteins are essential for bacterial survival, involved in a wide variety of processes, including energy generation, nutrient uptake, and cell wall biosynthesis. They maintain the integrity of bacterial membrane and orchestrate essential processes like proton-motive force generation and molecular transport [1–6]. YidC, a highly conserved membrane protein insertase, is a central facilitator of protein homeostasis in the membrane [7–13]. It enables the targeting, insertion and folding of a broad spectrum of proteins to the cell membrane, via both Sec-dependent and Sec-independent pathways [7,13–23]. YidC displays an astonishing substrate-multiplexing ability, spanning from small peptides up to multi-domain large proteins. Importantly, the insertion and assembly of multi-domain membrane proteins require the SecYEG translocon, highlighting the cooperative role of YidC and SecYEG in membrane protein biogenesis. In comparison, proteins of the YidC-only pathway are typically small membrane proteins and lack large periplasmic domains [12,13,15,24,25].

Apart from its function in protein biogenesis, YidC is also engaged in quality control to ensure that proteins inserted into the membrane are correctly folded. Experimental evidence supports a direct interaction between YidC and FtsH [26], while some studies suggest an indirect regulatory link [27]. Additionally, several review articles discuss the potential functional relationship between these proteins [28–30]. This partnership is thought to be critical for maintaining protein homeostasis and preventing the accumulation of dysfunctional proteins. However, the basis for this YidC-FtsH interaction remains unclear, and further functional and structural studies are needed to elucidate the molecular mechanisms.

Since YidC serves as a platform of membrane protein assembly and quality control in bacteria, it is an appealing target for novel antimicrobial strategies [31,32]. Small molecules binding critical functional regions, such as the hydrophilic groove or the “greasy slide” domain (see below), have been proposed to disrupt bacterial membrane protein folding and insertion, ultimately arresting bacterial growth [31–34]. This review focuses on the architecture of YidC, its role in membrane protein biogenesis and quality control, as well as its relevance as a potential drug target for next-generation antimicrobials.

## 2 Structure and function of YidC

**Fig. 1:**
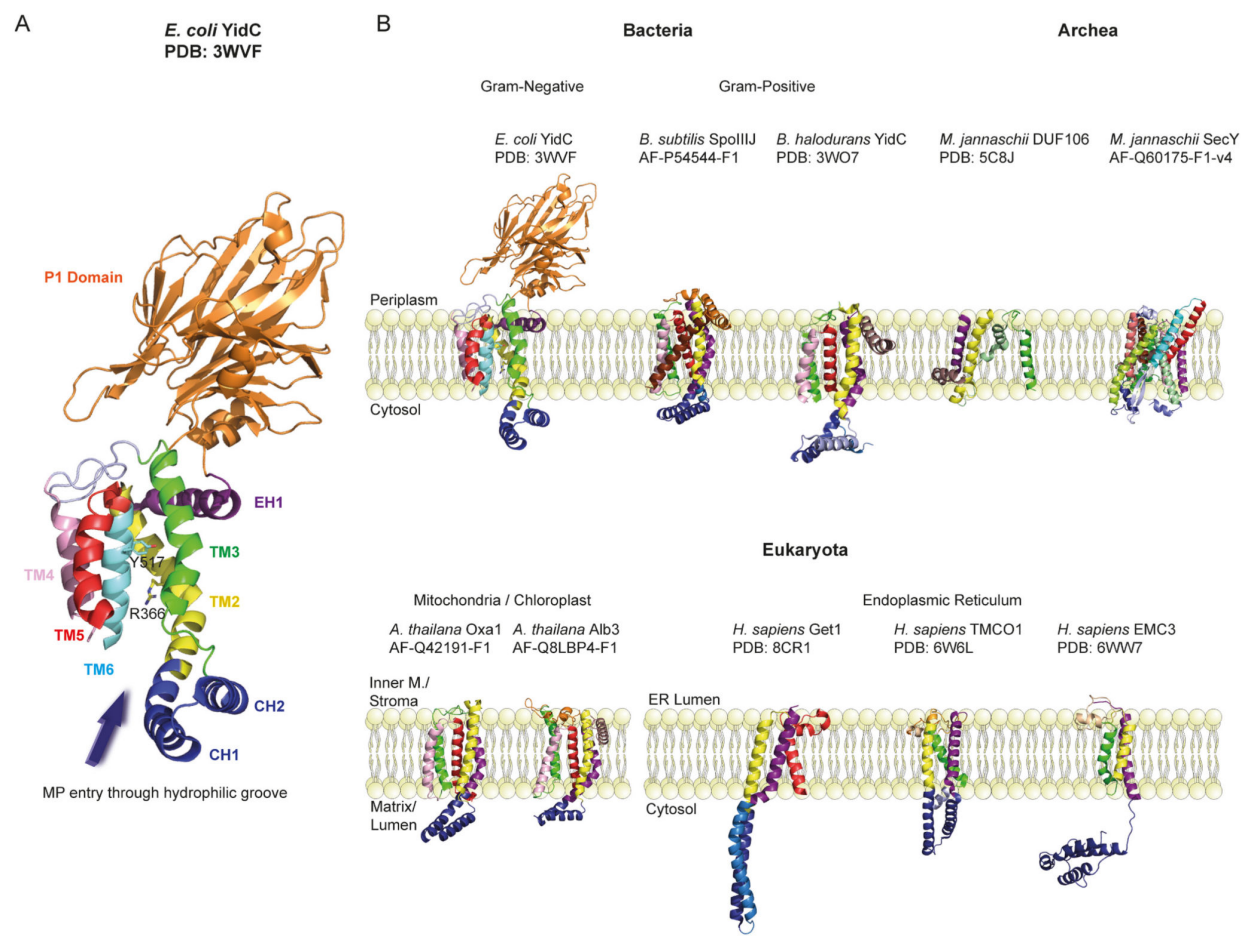
Structure and Evolution of YidC. A. Structure of YidC. EH1 (TM1), TM2–6 colored in purple, yellow, green, pink, red, cyan, respectively, P1 domain in orange color. The hydrophilic groove is highlighted with an arrow. B. Evolutionary Conservation and Structural Organization of YidC/Oxa1/Alb3 Insertases Family Proteins in the Membrane. *E. coli* YidC PDB: 3WVF, TM1–6 colored in purple, yellow, green, pink, red, cyan, respectively. Hydrophilic groove is between TM3 (green) and TM5 (red). (For interpretation of the references to color in this figure legend, the reader is referred to the web version of this article.)

The structural and functional characterization of YidC, primarily through X-ray crystallography and cryogenic electron microscopy (cryo-EM), has significantly advanced our understanding of its role as a membrane protein insertase [33–38]. Earlier studies resolved the *E. coli* YidC periplasmic domain through X-ray crystallography ([39], PDB: 3BS6; [40], PDB: 3BLC). Further insights were gained from the structure of *Bacillus halodurans* YidC form I and II (PDB: 3WO6 and 3WO7), which revealed the hydrophilic groove within the membrane core and provided a mechanistic model for Sec-independent membrane protein insertion [38]. The first complete structure of *E. coli* YidC, determined by X-ray crystallography (PDB: 3WVF), provided crucial insights into its conserved core architecture and was key in elucidating YidC’s function [33]. Subsequent structural studies corroborate YidC’s conserved architecture and its intrinsic structural flexibility; both are key to its multifaceted function in membrane protein biogenesis across phylogenetically diverse organisms (Fig. 1B) [41–46].

*E. coli* YidC is composed of six transmembrane helices (TM1-TM6), a large periplasmic domain (PD), two cytoplasmic loops (C1 and C2), and an amphipathic EH1 helix (TM1) (Fig. 1A). The transmembrane domain (helices TM1-TM5), the core of the protein, comprises a conserved hydrophilic groove, which is surrounded by TM3 and TM5 with contributions of the other transmembrane helices. This groove plays an important role in binding and stabilizing hydrophilic regions of membrane protein substrates. It serves as a first docking site for substrates before incorporation into the bilayer [32,33,36,37,47].

TM6, positioned adjacent to the hydrophilic groove (Fig. 1A), is required to maintain the structural integrity of the transmembrane region and ensures that dynamic rearrangements of YidC do not compromise its function [34]. The P1 region (Fig. 1A) within the periplasmic domain of YidC is required for Sec-dependent pathways by interacting with components of the SecYEG-SecDF-YajC-YidC holotranslocon (HTL) supercomplex, namely the periplasmic domains of SecD and SecF. These interactions are essential during the insertion of membrane proteins, facilitated by the proton motive force (PMF) and SecDF. The P1 domain also likely contacts polypeptides emerging from the SecYEG translocon. The transmembrane domain of YidC is suggested to aid in membrane protein insertion into the lipid bilayer during co-translational translocation; it supports folding of proteins by binding the transmembrane helices when they exit the lateral gate of SecY [20,35,48]. The P1 domain of YidC features a hydrophobic cleft that may interact with unfolded polypeptides in the periplasm, functioning as a molecular chaperone, while also stabilizing transmembrane segments through hydrogen bonds and mediating interactions with SecF for holo-translocon assembly [39,49]. Specifically, SecF binds to YidC residues 215 to 265 in the P1 domain, which consists of a short exposed α-helix and a long flexible loop [39,49]. This interaction, however, is not essential for YidC-Sec function, as a large deletion in the P1 domain prevents SecF binding, yet YidC continues to function in inserting Sec-dependent proteins, such as subunit A of F1Fo ATPase and M13 procoat derivatives [49]. Furthermore, the P1 domain is suggested to facilitate protein integration into the membrane in Sec-independent processes. Studies indicate that P1 may influence the kinetics of protein insertion and folding by directly interacting with the inserting protein chain to stabilize its structure [39,50]. Time-resolved fluorescence spectroscopy has shown that substrate binding to YidC induces backbone movements in the P1 domain, which retains its folded structure, further supporting an interaction of P1 with the inserting protein chain to stabilize and facilitate its membrane insertion [51].

Cryo-EM studies demonstrate that YidC binds the ribosome at the exit tunnel and dynamically aligns its hydrophilic groove and the ribosomal tunnel. YidC interacts with three main ribosomal contact points, which are also used by the SecYEG translocon: (i) helix 59 of the 23S rRNA, which is perturbed upon binding, (ii) a docking site near L23 and L29, commonly utilized by factors involved in cotranslational folding and translocation, and (iii) a weaker interaction site at L24 and helix 24 of the 23S rRNA [52]. The EH1 helix of YidC adopts a significantly different conformation during ribosome binding and co-translational protein insertion and possibly during post-translational protein insertion where EH1 is positioned at the membrane interface [51]. EH1 displacement towards the membrane core has been suggested to thin the lipid bilayer and thus facilitate nascent chain insertion [53]. Alternatively, EH1 may act as a mechanical lever, tilting TM3 and triggering the release of the nascent chain from the hydrophilic YidC groove into the lipid bilayer [34].

The cytoplasmic loops C1 and C2 of YidC (Fig. 1A) synergistically enhance its ribosome-binding capability [22,34,41]. The C2 loop is crucial for tight ribosome binding, optimally positioning YidC for nascent chain interaction, and forming a part of a composite ribosome-binding site with the C-terminus of YidC [22]. The C2 loop-ribosome interaction is modulated by nascent polypeptides [21]. In contrast, the C1 loop of YidC stabilizes nascent chains during bilayer insertion, supporting proper alignment with the ribosomal exit tunnel and protein folding. Thus, the C1 loop plays a role in downstream activities of YidC including substrate binding and insertion rather than direct ribosome binding [22,41]. The interplay between C1 and C2 loops facilitates the efficient transfer of the nascent polypeptide chain from the ribosomal exit tunnel into the lipid bilayer. This process ensures that hydrophobic regions integrate into the membrane while hydrophilic regions remain exposed to the cytoplasm or periplasm, thereby promoting proper membrane protein folding and assembly [34,53,54].

### 2.1 Structural and evolutionary conservation of YidC

Structural conservation of YidC in all domains of life underlines its biological importance [8,43,45] (Fig. 1B). Mitochondrial and chloroplast YidC homologs, Oxa1 and Alb3, both comprise a hydrophilic groove but have evolved specific characteristics to suit their functions [8,30,42,55–57]. For instance, Oxa1 contains a ribosome-tethering domain to facilitate co-translational assembly of respiratory chain supercomplexes [58–60]. Alb3 associates with the chloroplast signal recognition particle (cpSRP) to assemble photosystem complexes in thylakoid membranes [46,58].

These homologs aside, YidC also displays structural and functional homology to SecY [45], implying a shared evolutionary history. Both SecY and YidC share a similar core structure consisting of a membrane-spanning protein-conducting channel. The hydrophilic groove of YidC aligns with the hydrophilic funnel of SecY, suggesting a conserved mechanism for substrate binding, retention and lateral movement [45]. Both transporters have a three-helix bundle that forms this groove, with each transporter using this feature to translocate polypeptides across the membrane. This structural feature is essential for the transport of substrates through the hydrophobic membrane environment (Fig. 1B) [45]. The conserved features of the two halves of SecY suggest that their common ancestor was an antiparallel homodimeric channel, with each half contributing to the formation of the protein-conducting pathway. This dimeric architecture is also observed in YidC, supporting the idea that both transporters evolved from a shared ancestor with a similar structural and functional mechanism [45]. However, SecY is involved in the transport of membrane proteins and secretory proteins, whereas YidC is employed only in the insertion of hydrophobic membrane proteins, indicating evolutionary divergence from an ancestral mechanism [45].

The archaeal protein DUF106 from *M. jannaschii* (Mj0480) also possesses a YidC-like fold. Thus, evolution involving an ancestral membrane protein insertase prior to the branching of bacteria, archaea, and eukaryotes has been suggested [43] (Fig. 1B). However, it remains unclear whether the ancestral protein that gave rise to insertases had a 3 TM segment core [8], as seen in archaeal and ER insertase homologs, or a 5 TM segment core [43,59], as found in bacterial, mitochondrial, and chloroplast homologs while the 5 TM architecture is likely a later adaptation in the course of evolution. This distinction may be crucial for understanding the evolutionary origins and functional diversification of these insertase proteins. This structural and evolutionary conservation is consistent with the notion that YidC is a central facilitator of membrane protein biogenesis in a wide variety of cellular contexts.

### 2.2 Role of YidC in bacterial membrane protein insertion, folding, and quality control

YidC functions as both an insertase and a chaperone in membrane protein biogenesis. By acting via Sec-dependent and Sec-independent mechanisms, YidC mediates insertion, folding, and complex assembly of membrane proteins, and may be involved in degradation of misfolded proteins [11,12,16]. The insertase and chaperone functions of YidC appear to be interdependent, and no study has evidently separated them functionally. However, it has been shown that, at least in the case of LacY insertion, LacY is inserted independently of YidC, while YidC is required for LacY folding [60]. Both roles contribute to cellular homeostasis by preventing the accumulation of misfolded membrane proteins [11,12,27,61,62].

Early studies indicate that YidC interacts directly with the emerging chain of MtlA, a Sec-dependent substrate, at the protein-lipid interface [63]. YidC is able to accommodate at least the first two transmembrane segments of MtlA, even though the nascent chain is long enough to allow additional segments to insert into the membrane. This suggests that YidC forms a contiguous integration unit with the SecYEG translocon, functioning as an assembly site for polytopic membrane proteins and mediating the formation of helix bundles before their release into the membrane [63]. This interaction underscores YidC’s function as a chaperone, similar to TRAM in the ER membrane, where TRAM assists in the proper integration of nascent proteins into the membrane [59].

YidC works together with other factors to facilitate membrane protein insertion, particularly through its interaction with MPIase, a glycolipozyme that plays a crucial role in the integration of membrane proteins [64,65]. MPIase is essential for the insertion of both Sec-dependent and Sec-independent substrates via YidC, and its depletion leads to defects in protein insertion [66,67]. While YidC is not essential for the insertion of proteins like Pf3-Lep, it significantly accelerates the process when substrate levels are higher. This suggests that YidC’s role becomes more prominent under conditions of increased substrate concentration [13]. Moreover, YidC and MPIase cooperate during membrane protein biogenesis by enhancing MPIase activity, helping to insert membrane proteins more efficiently [68]. The upregulation of MPIase in response to YidC depletion further supports this, as the cell compensates for the loss of YidC by increasing MPIase levels [13,69]. Together, MPIase and YidC ensure the proper integration of membrane proteins, particularly during stress conditions when the cell faces challenges in maintaining membrane integrity and protein function.

Beyond its insertase role, YidC stabilizes nascent proteins during their folding, prevents protein aggregation, and ensures that only properly folded proteins are retained in the membrane [70,71]. YidC’s interactions with quality control proteins that include FtsH, may enable it to identify and degrade misfolded proteins, maintaining a cell membrane virtually free of defective proteins [27,61,62].

Loss or depletion of YidC significantly impacts cellular homeostasis and critically attenuates biogenesis of essential membrane proteins that drive energy transduction and membrane integrity [14,27,62]. YidC loss of function results in decreased proton-motive force which is likely linked to YidC being required for insertion of FoC (subunit C of ATPase) and C ring assembly and ATP synthesis, severely impacting cellular metabolism and growth [27,62]. Finally, YidC depletion triggers upregulation of HflK, HflC, GroEL, DnaK, PpiD, OppA, UspA, PspA and other proteins, implicated in cellular stress responses (Table 1) [9,27,62]. Induction of stress responses including the phage shock protein (Psp) response upon YidC depletion highlights cellular efforts to maintain inner membrane integrity [9].

### 2.3 Sec-dependent and Sec-independent pathways of YidC in bacteria

**Fig. 2:**
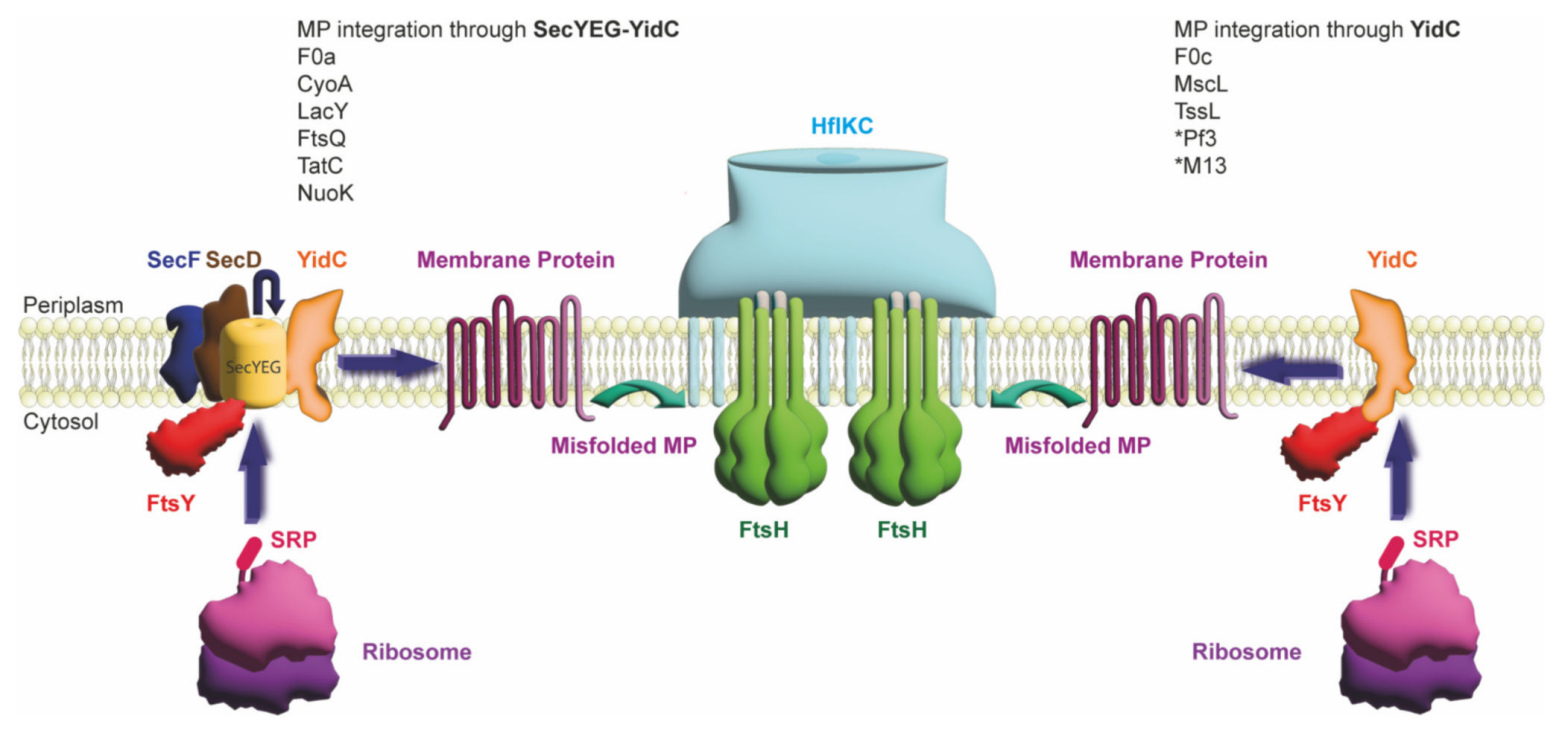
Bacterial Membrane Protein Translocation via Sec-dependent and Sec-Independent pathways and hypothetical bacterial membrane protein quality control mechanism. *Pf3 and *M13 are too small for co-translational SRP recognition; instead, they are recognized post-translationally and integrated into the membrane through YidC.

YidC-driven translocation, folding, and complex formation of membrane proteins occur via two distinct pathways: Sec-dependent and Sec-independent [16,23,24]. In the Sec-independent pathway, YidC integrates small membrane proteins, including F1Fo ATP synthase subunit c [55,72], M13 procoat protein [7], Pf3 coat protein [15], MscL [73] and TssL [74] into the membrane. The hydrophilic groove transiently accommodates hydrophilic substrate regions, while the “greasy slide” enables lateral movement of hydrophobic regions into the bilayer [75,76] (Fig. 2).

In the Sec-dependent pathway, YidC works with SecYEG and the SecYEG-SecDF-YajC holo-translocon complex to integrate complex substrates with hydrophilic and hydrophobic regions [20,35,48]. Their collaboration ensures the correct folding and membrane insertion of vital energy-transducing complexes such as F1Fo ATP synthase subunit a [73,77], cytochrome bo oxidase [17,78], and NADH dehydrogenase [61]. The interaction between YidC and SecYEG in the Sec-dependent pathway corroborates the fact that YidC can accommodate very diverse substrates (Table 1). YidC efficiently supports the insertion of both small hydrophobic proteins and large multi-domain complexes into the membrane. YidC thus plays a central role in maintaining membrane protein homeostasis and critically contributes to the overall integrity and function of the cell.

### 2.4 Substrates of YidC

YidC supports the biogenesis of substrates ranging from small hydrophobic peptides to large, multi-domain membrane protein complexes (Table 1). Proteomics studies revealed that between 17 % and 32 % of bacterial membrane proteins rely on YidC for proper assembly [6,79]. This remarkably wide range of proteins affected by YidC depletion points to a large number of YidC substrates, emphasizing YidC’s critical function in bacterial protein biogenesis [75,80].

Key energy-transducing complexes, such as F1Fo ATP synthase, cytochrome bo oxidase, and NADH dehydrogenase, depend on YidC to support proper integration and assembly of specific subunits of these complexes in the lipid bilayer [17,61,77,78,81]. However, it is important to note that YidC is often responsible for only one of the multiple subunits in these energy-transducing protein complexes. F1Fo ATP synthase subunit c is inserted via a YidC-driven, Sec-independent pathway, utilizing the hydrophilic groove and greasy slide to achieve correct transmembrane topology, and assembly of the c-ring occurs subsequently to form the functional rotor structure [18,72,82]. YidC also enables the translocation of small nonpolar proteins like M13 and Pf3 coat proteins [7,15]. These substrates fully depend on YidC for insertion and have in common that they consist of one or two transmembrane helices linked by a short loop. Membrane proteins with large hydrophilic domains require Sec-dependent translocation [16] (Fig. 2). Examples for such larger membrane proteins are FtsQ [14,83] and lactose permease [60], which depend on collaboration between YidC and SecYEG for correct membrane integration and folding. In the SecYEG-YidC collaboration, YidC is suggested to ensure lateral release and proper folding of the transmembrane domains while SecYEG is critical for translocation of hydrophilic regions [21,23]. Additionally, proteins with more than two transmembrane helices generally depend on the Sec translocon for proper membrane integration, while YidC is sufficient for inserting small single or double transmembrane helix proteins [75] (Fig. 2).

## 3 YidC in bacterial membrane protein quality control

Crosstalk between YidC and FtsH is implicated in bacterial membrane protein quality control [26,28,29,48]. YidC mediates the insertion and folding of nascent membrane proteins, while FtsH degrades misfolded or unassembled proteins, preventing their accumulation [26]. The cooperation between YidC and FtsH guarantees the integrity of the membrane resident functional proteins and prevents cellular dysfunction due to the presence of malfunctioning proteins [26].

FtsH is a hexameric protease with a transmembrane domain and ATPase - protease domains located in the cytoplasm. FtsH can associate with HflK and HflC, regulatory proteins that “vault” around FtsH. HflK and HflC have been suggested to stabilize the hexameric structure of FtsH and regulate substrate access [84–86]. Recent structural studies describe the FtsH-HflK-HflC complex as having a bell-shaped or nautilus-like architecture. This complex plays a crucial role in proteostasis by selectively degrading non-functional membrane proteins while allowing functional proteins to remain intact. Additionally, both YidC [87,88] FtsH-HflKC [86] are thought to play a role of lipid scramblase, an enzyme that facilitates the translocation of lipids between membrane leaflets, highlighting the multifaceted roles of the FtsH-HflKC and YidC in membrane protein quality control [86].

Based on photo-crosslinking and chemical crosslinking proteomic analyses, YidC, FtsH, and the HflKC have been suggested to associate with each other [26,27,89,90]. It appears that YidC operates in close proximity to FtsH within the trajectory of membrane protein biogenesis [26]. A potential direct protein – protein interaction between YidC and FtsH might ensure that correctly folded proteins are retained, and incorrectly folded proteins are degraded in the membrane. However, to date, there is no structural data reported showing a physical interaction between YidC and FtsH. Studying this interaction biochemically and structurally is critical to obtain a better understanding of this crucial step in membrane protein quality control.

A YidC-FtsH complex would present a highly selective machinery for protein integrity linking folding, assembly, and degradation – processes crucial for bacterial viability. Disruption of YidC or FtsH both results in accumulation of misfolded proteins, stress response activation, and loss of membrane protein function [26,48]. Future studies of the structural and functional interplay and interaction of the YidC-FtsH are required to bridge a key gap in our understanding of bacterial membrane proteostasis. Moreover, it offers a promising avenue for the development of novel antimicrobial strategies targeting bacterial quality control systems.

## 4 YidC as a potential target for novel antibiotics

YidC has been suggested to be a target to develop new antibiotics [31]. In fact, small-molecule inhibitors such as carvacrol, eugenol, Cpd36 and Cpd46 have been identified that block YidC’s essential function in bacterial membrane protein folding and insertion, and that lead to an arrest of bacterial growth [32]. The conserved hydrophilic groove of YidC could represent an appealing target for drug binding, allowing selective interference in substrate binding without major structural changes [31–33].

The natural compounds carvacrol and eugenol have demonstrated inhibitory activity against YidC. These inhibitors block bacterial growth and additionally sensitize bacteria to other antibiotics, suggesting their application in combination therapies [32]. Synthetic analogs, based on celecoxib (Cpd36, Cpd46), inhibit YidC’s activity in antibiotic-resistant bacteria such as *Staphylococcus aureus*, which expresses YidC2, a homolog of YidC that plays a similar role in membrane protein insertion and folding [32]. While small-molecule inhibitors like Carvacrol and Eugenol have been suggested to target YidC, current evidence indicates that their primary mechanism of action is through membrane disruption and ATPase inhibition, rather than direct binding to YidC [31]. In contrast, Cpd36 has been shown to directly bind to the hydrophilic groove of *S. aureus* YidC2. Mutagenesis studies have pinpointed key residues such as Y188, L240, P73, P139, and Y79 as critical contact points for Cpd36 binding, confirming a direct inhibitory interaction. Thermal stability shift assays further support this finding, demonstrating a significant stabilization of YidC2 upon Cpd36 binding, distinguishing it from membrane-disrupting agents like Carvacrol and Eugenol [32].

Antisense RNA technology has been used to inhibit expression of YidC in bacteria. This resulted in decreased bacterial growth and again in enhanced susceptibility to antimicrobials validating the approach [31].

Despite these promising developments, challenges remain. In particular, ensuring bacterial selectivity without targeting eukaryotic YidC homologs remains a major challenge [32]. Potentially, more high-resolution structures of functional YidC complexes could enable the design of more selective inhibitors targeting other protein-protein interaction sides. Combination therapies of such YidC inhibitors with other antimicrobials could prevent bacterial resistance and improve efficacy.

## 5 Discussion and conclusions

YidC is a master regulator of membrane protein insertion, folding and complex assembly. Its capacity to function in both Sec-dependent and Sec-independent pathways enables it to process a wide spectrum of substrates spanning from small proteins with single transmembrane segments to very large multi-domain and multi-subunit membrane protein systems. YidC’s substrates function in energy metabolism and transport, and consequently YidC is essential for cellular homeostasis.

Besides its function as a membrane protein insertase and chaperone, YidC plays a critical role in membrane protein quality control. Through a largely uncharacterised cooperation with the protease FtsH, YidC functions in the recognition of misfolded proteins and their degradation contributing to membrane homeostasis. The molecular mechanisms of the YidC and FtsH structural and functional interaction are not well understood, and further investigations are required to advance our understanding. Learning the role of YidC in relation to other pathways involving the FtsH protein and the FtsH-HflK-HflC complex will have significant implications for our views on how bacteria maintain their membranes under normal and stress conditions.

YidC’s conserved structure and functional roles renders it a target for antibiotics. Potential molecules that bind to known functionally important sites of YidC, including its hydrophilic groove, are suggested to abolish membrane protein biogenesis and inhibit bacterial growth. Nevertheless, challenges exist to generate inhibitors with the required specificity for bacterial YidC rather than binding and inhibiting also YidC’s homologous human proteins.

In conclusion, YidC has surprisingly versatile roles in protein trans-location and quality control. Studies of YidC structure and function provide insights into its functional effects on bacterial membranes. Future research into YidC’s mechanisms of action as well as its functional interplay with other membrane proteins will enhance our knowledge of bacterial physiology and enable the discovery of urgently needed antibacterial drugs to counter the global threat of antibiotic resistance.

## Figures and Tables

**Table 1:** YidC-associated proteins and their biological importance. Proteins associated with YidC are listed with their relationships to YidC. Biological roles of YidC substrates are described.

YidC-related proteins	Protein full name	Relation with YidC	Biological importance	References
Pf3	Phage Shock Protein Pf3	YidC-Dependent Membrane Integration	Structural proteins of bacteriophages	Samuelson et al., 2000; Polasa et al., 2023
M13	M13 Procoat Protein	YidC-Dependent Membrane Integration	Structural proteins of bacteriophages	Samuelson et al., 2000; Chen et al., 2002
F0c	F1FO ATP Synthase Subunit c	YidC-Dependent Membrane Integration	Essential for ATP synthase function	Scotti et al., 2000; Bloois et al., 2004
MscL	Mechanosensitive Channel of Large Conductance	YidC-Dependent Membrane Integration	Mechanotransduction processes	Facey et al., 2007; Pop et al., 2009
TssL	Type VI Secretion System Subunit L	YidC-Dependent Membrane Integration	Involved in bacterial secretion	Aschtgen et al., 2012;
F0a	F1FO ATP Synthase Subunit a	Membrane Integration through SecYEG-YidC Pathway	Crucial for ATP generation	Yi et al., 2004; Kol et al., 2009
CyoA	Cytochrome Bo Oxidase Subunit II	Membrane Integration through SecYEG-YidC Pathway	Part of aerobic respiration	Celebi et al., 2006; Plessis et al., 2006
LacY	Lactose Permease	Membrane Integration through SecYEG-YidC Pathway	Involved in sugar metabolism	Zhu et al., 2013
FtsQ	Filamentation Temperature-Sensitive Protein Q	Membrane Integration through SecYEG-YidC Pathway	Critical for bacterial cell division	Laan et al., 2000; Urbanus et al., 2001
TatC	Twin-Arginine Translocase Subunit C	Membrane Integration through SecYEG-YidC Pathway	Key component of twin-arginine transport	Zhu et a., 2012
NuoK	NADH: Quinone Oxidoreductase Subunit K	Membrane Integration through SecYEG-YidC Pathway	Supports energy metabolism	Price and Driessen, 2009
Lep	Leader peptidase	Involved in signal peptide processing with YidC	Signal peptide cleavage	Houben et al., 2000
SecY	Sec Translocase Subunit Y	Membrane Integration through SecYEG-YidC Pathway	Part of SecYEG holotranslocon complex	Houben et al., 2002
SecE	Sec Translocase Subunit E	YidC-Dependent Membrane Integration	Part of SecYEG holotranslocon complex	Yi et al., 2003
SecG	Sec Translocase Subunit G	Co-purified wtih YidC	Part of SecYEG holotranslocon complex	Petriman et al., 2018; Bloois et al., 2008
SecF	Holotranslocon Subunit F	Part of HTL, co-purified with YidC	Involved in translocation and insertion	Schulze et al., 2014; Bloois et al., 2008
SecD	Holotranslocon Subunit D	Part of HTL, co-purified with YidC	Involved in translocation and insertion	Schulze et al., 2014; Bloois et al., 2008
YajC	Holotranslocon Subunit YajC	Part of HTL	Involved in translocation and insertion	Schulze et al., 2014
SecA	Protein Translocase Subunit A	Interacts with HTL including YidC	Involved in translocation and insertion	Schulze et al., 2014
Ffh	SRP Component Ffh	Binds YidC C1 loop for protein integration via SRP	Targets proteins to membrane	Bloois et al., 2004
L23 & L24	Ribosomal Large Subunit Proteins L23 and L24	YidC binds L23 and L24 for co- translational protein insertion	Ribosome-associated nascent chain protection	Bloois et al., 2004
PBPs	Penicillin-Binding Proteins	Assembly of PBP complex depending on YidC	Cell wall synthesis, antibiotic resistance	Borges et al., 2015
MalF & MalKG	Maltose Transporter Subunits MalF and MalKG	Assembly of the complex depending on YidC	Part of sugar transport system	Wagner et al., 2008
FtsH	Filamentation Temperature-Sensitive Protein H	Co-purified with YidC, upregulated in YidC depletion	Involved in membrane protein quality control	Bloois et al., 2008; Wickström et al., 2011; Akkulak et al., 2024
HflK & HflC	High Frequency Lysogenization Proteins HflK and HfIC	Co-purified with YidC, upregulated in YidC depletion	Involved in membrane protein quality control	Bloois et al., 2008; Wickström et al., 2011; Akkulak et al., 2024
QmcA	Quality Control Membrane Protein A	Upregulated in YidC depletion	Supports membrane protein quality control	Wickstörm et al., 2011
PspA	Phage Shock Protein A	Upregulated in YidC depletion	Proton motive force stabilization	Bloois et al., 2005; Wang et al., 2010
TF	Trigger Factor	Upregulated in YidC depletion	Ensures proper folding of membrane proteins	Wang et al., 2010
GroEL & GroES	GroEL and GroES Chaperonins	Upregulated in YidC depletion	Stress response chaperones	Wang et al., 2010; Wickstörm et al., 2011
DnaK	Heat Shock Protein 70 (HSP70)	Upregulated in YidC depletion	Stress response chaperones	Wickstörm et al., 2011
DnaJ	Heat Shock Protein DnaJ	Upregulated in YidC depletion	Stress response chaperones	Wang et al., 2010; Price et al., 2010
HtpG	Heat Shock Protein G	Upregulated in YidC depletion	Stress response chaperones	Price et al., 2010
DsbA	Disulfide Bond Isomerase A	Upregulated in YidC depletion	Confers multidrug resistance	Wang et al., 2010
ppiA	Peptidyl-Prolyl Cis-Trans Isomerase A	Upregulated in YidC depletion	Protein folding and peptide isomerization	Wang et al., 2010
ppiD	Peptidyl-Prolyl Cis-Trans Isomerase D	Upregulated in YidC depletion	Membrane protein folding chaperone	Wickstörm et al., 2011
OmpC	Outer Membrane Protein C	Upregulated in YidC depletion	Maintaining outer membrane permeability	Wang et al., 2010
OppA	Oligopeptide Transport Periplasmic Protein A	Upregulated in YidC depletion	Facilitating oligopeptide transport	Wickstörm et al., 2011
ClpB	Caseinolytic Peptidase B	Upregulated in YidC depletion	Refolding aggregated proteins under stress	Wang et al., 2010; Price et al., 2010
FimC	Fimbrial Chaperone Protein	Upregulated in YidC depletion	Fimbrial assembly	Wang et al., 2010
